# Homologous PB1 gene promotes the replication efficiency of avian influenza H7N4 candidate vaccine virus

**DOI:** 10.1111/irv.12954

**Published:** 2022-01-17

**Authors:** Liqi Liu, Zi Li, Jianfang Zhou, Jia Liu, Xiyan Li, Weijuan Huang, Ning Xiao, Dayan Wang

**Affiliations:** ^1^ Chinese National Influenza Center National Institute for Viral Disease Control and Prevention, China CDC Beijing China

**Keywords:** avian influenza H7N4 virus, candidate vaccine virus, polymerase basic protein 1, reverse genetics

## Abstract

**Background:**

The first and only case of human infection with the avian influenza A (H7N4) virus in China emerged in 2018. The H7N4 virus was distinct from previous H7N9 viruses and raised public concerns. Therefore, developing a suitable H7N4 candidate vaccine virus (CVV) remains crucial for potential pandemic preparedness.

**Methods:**

We constructed a reassortant virus with a (6 + 2) genome composition, then introduced the polymerase basic protein 1 (PB1) from a wild‐type virus to develop a (5 + 3) reassortant virus through reverse genetics. We performed whole‐genome sequencing to confirm the genome stability, assessed the growth ability in MDCK cells, and analyzed virus antigenicity using hemagglutination inhibition assays. Subsequently, the effect of homologous PB1 on polymerase activity, viral protein yield, and pathogenicity was assessed.

**Results:**

The (5 + 3) virus harbouring the homologous PB1 gene exhibited significantly improved growth characteristics, higher viral protein yield, and polymerase activity than the (6 + 2) virus. After successive passage in embryonated eggs, glutamic acid (E) substituted glycine(G) at position 218 (H3 numbering) in the hemagglutinin (HA) gene of both (5 + 3) and (6 + 2) viruses. The substitution improved the growth of the (6 + 2) virus but exhibited no significant effect or alteration on the antigenicity of the (5 + 3) virus. Moreover, the (5 + 3) virus exhibited low pathogenicity in chickens and ferrets.

**Conclusion:**

Homologous PB1 of the H7N4 virus improves the growth ability while sustaining low pathogenicity. Collectively, the gene composition of the (5 + 3) reassortant virus is a suitable H7N4 CVV for potential pandemic preparedness.

## 1 INTRODUCTION

Influenza viruses are highly contagious respiratory pathogens that threaten public health worldwide. Besides seasonal epidemics, influenza A viruses potentially influence the emergence of future pandemics whenever novel or avian influenza virus develops efficient human‐to‐human transmission and spreads globally. The first recognized human infection of highly pathogenic avian influenza A (H5N1) virus, previously confined to poultry, emerged in 1997 during a poultry outbreak in Hong Kong.^1^ Since then, various avian influenza virus strains have infected humans, including H5N6, H7N9, H9N2, and H10N8, causing significant public health concerns.^2^ In 2018, the first case of human infection with avian influenza A (H7N4) virus, causing severe pneumonia and acute respiratory distress, emerged in China.^3^ Genome assembly showed 8 consensus gene segments of H7N4 from the throat swab (designated A/Jiangsu/1/2018, designated JS‐Hu in this article), but the virus was not successfully isolated from the patient samples.^4^ The JS‐Hu genes are closely related to the influenza virus, A/Chicken/Jiangsu/1/2018 (JS‐Ck, H7N4), which were eventually isolated from chickens fed by the patient.^4^ However, the H7N4 viruses were genetically and antigenically distinct from previously reported H7N9 viruses, which caused persistent and severe human infections in previous years.^4^

This prevalence of avian influenza viruses has raised great concerns regarding potential pandemics and warrants the developing corresponding vaccine strains for pandemic preparedness. As part of an influenza pandemic preparedness program for the novel H7N4 virus, the World Health Organization (WHO) recommended the JS‐Ck‐like virus as a candidate vaccine virus (CVV) based on the current antigenic, genetic, and epidemiologic data.^5^

Vaccines are the most potent and effective tool for public health. Owing to the lack of universal influenza vaccines, CVV development for specific influenza strains remains crucial. Thus, selecting high‐yield CVVs is critical for large‐scale pre‐pandemic vaccine production. The reverse genetics (RG) method accurately controls the genetic composition of the reassortant viruses by an eight plasmid‐based rescue system. This technique yields viral (6 + 2) genotype reassortant ‘seeds’ comprising the hemagglutinin (HA) and neuraminidase (NA) surface genes derived from wild‐type (WT) epidemic viruses while other six “internal genes” are from the high yield A/Puerto Rico/8/1934(PR8) virus. However, previous studies showed that the frequency of high yield reassortant viruses derived from the WT homologous polymerase basic protein 1 (PB1) gene is high, and the PB1 segment from WT virus might enhance the replication efficiency of CVVs.^6^, ^7^, ^8^ These reassortant viruses containing HA, NA, and PB1 from the WT combined with other genes from PR8, were designated as (5 + 3) reassortant viruses for selecting high yield CVVs.^9^ In contrast, some studies reported that PB1 neither drives selecting the suitable virus nor selects the best yielding virus for vaccine production.^10^, ^11^ Moreover, PB1 affects the replication capacity and pathogenicity of the reassortant virus.^12^, ^13^

An effective CVV should have high replication capacity and antigenic consistency with the prototype strain, with low pathogenicity.^14^ This study constructed two viral reassortants with (6 + 2) and (5 + 3) genomes generated from the JS‐Ck virus using RG. The purpose was to develop a suitable H7N4 CVV that meets vaccine requirements. The only difference between (6 + 2) and (5 + 3) viruses was the PB1 gene segment from PR8 or JS‐Ck H7N4. We performed serial assessments to understand the effect of homologous PB1 on H7N4 CVV. This is the first known report on the development and evaluation of CVVs for H7N4 subtype influenza virus.

## 2 METHODS

### 2.1 Generation of reassortant viruses by RG

HA, NA, and PB1 genes were synthesized (GenScript, NJ, USA) based on the H7N4 virus JS‐Ck or JS‐Hu, and cloned into vector pHW2000 plasmid. Reassortant viruses [(6 + 2) or (5 + 3)] were rescued as described previously.^15^ The first‐generation of rescued viruses were designated as V1E1 [rescued in Vero (V) cells followed by one passage in eggs (E)] and wholly sequenced using next‐generation sequencing (NGS) on the Illumina NextSeq 550 Sequencing System platform to confirm the origin of genes.

### 2.2 Stability of reassortant viruses

For the stability test, viruses were consecutively passaged six times through specific‐pathogen‐free (SPF) embryonated eggs following the WHO guidelines.^14^ Each generation virus was stored at −80°C until further use. All viruses of each generation were sequenced by NGS and analyzed using the BioEdit (Version 7.1.3.0) software.

### 2.3 Growth kinetics assay

MDCK cells were used to investigate growth characteristics of different viruses as described previously.^15^ Cells were infected with viruses at 0.001 multiplicity of infection (MOI), and supernatants were collected at 24, 48, 72, and 96 h post‐infection. The TCID_50_ hemagglutination assay determined the viral infectivity titers.

### 2.4 Assay for polymerase activity

The polymerase activity was measured by minigenome transfection and virus infection, as previously described, with slight modifications.^13^, ^16^ HEK 293T cells were co‐transfected with four pHW2000 plasmids containing the viral coding segments PB1, PB2, PA, and NP for minigenome transfection. The plasmids also contained the reporter plasmid pHH21‐NP‐Luc (the open reading frame of the luciferase gene, flanked by the 5′and 3′ noncoding regions of the NP gene of PR8) and internal control plasmid pGL4.73 (Promega, WI, USA; encoding Renilla luciferase) co‐transfected using the SuperFect Transfection Reagent (Qiagen, Hilden, Germany). The background control was three plasmids lacking the PB1 subunit. At 24 h post‐transfection, cell lysates were subsequently assayed for luciferase activities with the dual‐luciferase reporter assay system following the manufacturer's instructions (Promega, WI, USA). For virus infection, Vero cells were transfected with the reporter plasmid pHH21‐NP‐Luc and internal control plasmid pGL4.73. At 12 h post‐transfection, the transfection media were removed, and cells were infected with the virus (MOI = 1). The negative control was virus‐free culture media. The cell lysates were collected 24 h post‐infection to measure the polymerase activity. The pGL4.73 Renilla luciferase expression was the internal control for normalizing transfection efficiency. The polymerase activity containing PR8 minigenome or virus was standardized to 100%. Luciferase activities were measured on the Envision Multilabel Plate Reader (PerkinElmer, MA, USA).

### 2.5 Plaque formation assay

MDCK cells were used to determine the plaque formation, and cells were infected with 0.01 MOI. The assay was performed according to our previous report.^17^

### 2.6 Hemagglutination assay and hemagglutination inhibition (HI) assay

Hemagglutination and HI assays were performed as described in the WHO Manual for Laboratory Diagnosis and Virological Surveillance of Influenza using 1% turkey erythrocytes.^18^

### 2.7 Purification and quantification of viral proteins

Viruses were purified from allantoic fluids through sucrose (Solarbio, Beijing, China) gradient centrifugation.^17^ Total protein was determined by a BCA protein assay kit (Thermo Fisher Scientific, MA, USA) as previously described.^17^ The protein was digested using PNGase F (New England Biolabs, MA, USA), separated by 4%–20% SDS gel electrophoresis (Bio‐Rad, CA, USA), and visualized by Coomassie staining. The Image lab software 6.0. (Bio‐Rad, CA, USA) quantified the HA protein. The proportion of HA was determined using the following equation: HA1 + HA2/HA1 + HA2 + NP + M as previously described.^17^

### 2.8 Ferret study

Ferrets (*Mustela putorius furo*), aged 4‐months, were obtained from Wuxi Sangosho Biotechnology Co., Ltd. (Jiangsu, China). Routine HI tests prescreened sera from ferrets to confirm the absence of antibodies to epidemic human influenza and test viruses. Before infection, ferrets were randomly selected and housed individually in isolation units with free access to food and water.

For antisera preparation, ferrets were infected with reassortant viruses through intranasal inoculation (500 μl per nostril). Blood samples were collected 14 days post‐inoculation (dpi) and tested by HI assays to determine the antibody titer.

Pathogenicity assessment tests were conducted according to the WHO guidelines.^14^ Briefly, 4‐month‐old ferrets (*n* = 4) were intranasally inoculated with 10^6^ TCID_50_ of the test virus in 1‐ml sterile phosphate‐buffered saline (PBS), and tissue samples were collected as previously described.^19^

### 2.9 Intravenous pathogenicity index in chickens

We assessed the intravenous pathogenicity index (IVPI) in SPF chickens to verify the virus pathogenicity. SPF chickens were purchased from Beijing Boehringer Ingelheim Vital Biotechnology Co., Ltd (Beijing, China). Meanwhile, the IVPI was conducted following the recommendation of the World Organization for Animal Health (OIE).^20^ Viruses with IVPI values below 1.2 were considered to have low pathogenicity in chickens.

### 2.10 Statistical analysis

Quantitative data are averages of three independent experiments and are expressed as means ± SD. Results were analyzed using the GraphPad Prism version 5.0 (GraphPad Software, Inc., CA, USA). A P‐value of <0.05 was considered statistically significant.

## 3 RESULTS

### 3.1 Stability of different reassortant viruses

The first‐generation rescued virus RG (6 + 2) JS‐Ck (V1E1, JS‐Ck62G) and RG (5 + 3) JS‐Ck (V1E1, JS‐Ck53G) were sequenced. The virus gene sequences were identical to the sequences that carried by plasmids used for constructing the viruses. The only difference between the two viruses (JS‐Ck62G and JS‐Ck53G) was that the PB1 gene was originated from PR8 and WT JS‐Ck, respectively.

The rescued V1E1 virus was propagated in chicken embryonated eggs for additional six passages to determine the genetic stability of the HA, NA, and PB1 genes. These were designated as V1Ex (x is the number of passages). All viruses of different passages were sequenced and compared with respective V1E1. Sequence analyses revealed no mutation in the NA and PB1 gene. However, one variation (from Glycine (G) to Glutamic acid (E)) was found at position 218 (H3 numbering) in the HA gene of both RG viruses. The G substitution with E in HA appeared in the V1E6 of the (6 + 2) virus (JS‐Ck62E) and the V1E4 of the (5 + 3) virus (JS‐Ck53E). The RG (6 + 2) JS‐Hu was rescued and propagated in embryonated chicken eggs six times to understand whether the substitution was prone in JS‐Hu virus. Interestingly, V1E4 had the same substitution at the same loci of the HA gene in JS‐Hu.

JS‐Ck62E and JS‐Ck53E were propagated in embryonated eggs six additional times to understand the stability of the mutant viruses. The results demonstrated no additional mutation in HA, NA and PB1 gene sequences. Besides, the stability of the mutant virus harbouring 218E was superior to the 218G virus. The genetic origins of the reassortant viruses are listed in Table 1.

**TABLE 1:** List of reassortant viruses generated and utilized in this study

Wild‐type virus strain	Generated virus by RG ^a^	Passage history ^b^	Genotype	Abbreviation
A/Chicken/Jiangsu/1/2018 (JS‐Ck, H7N4)	RG(6 + 2) JS‐Ck	V1E2	HA (218G) ^c^ and NA from wild virus JS‐Ck	JS‐Ck62G
		V1E6	HA (218E) and NA from wild virus JS‐Ck	JS‐Ck62E
	RG(5 + 3) JS‐Ck	V1E2	HA (218G), NA and PB1 from wild virus JS‐Ck	JS‐Ck53G
		V1E4	HA (218E), NA and PB1 from wild virus JS‐Ck	JS‐Ck53E
A/Jiangsu/1/2018 (JS‐Hu, H7N4)	RG(6 + 2) JS‐Hu	V1E2	HA (218G) and NA from human virus JS‐Hu	JS‐Hu62G
		V1E4	HA (218E) and NA from wild virus JS‐Hu	JS‐Hu62E

*Note*:

^a^ RG: reverse genetics;

^b^ V1Ex, initially rescued in Vero cells followed by *x* passages growth in eggs;

^c^ H3 numbering was used.

### 3.2 Growth characteristics of different reassortant viruses

**FIGURE 1:**
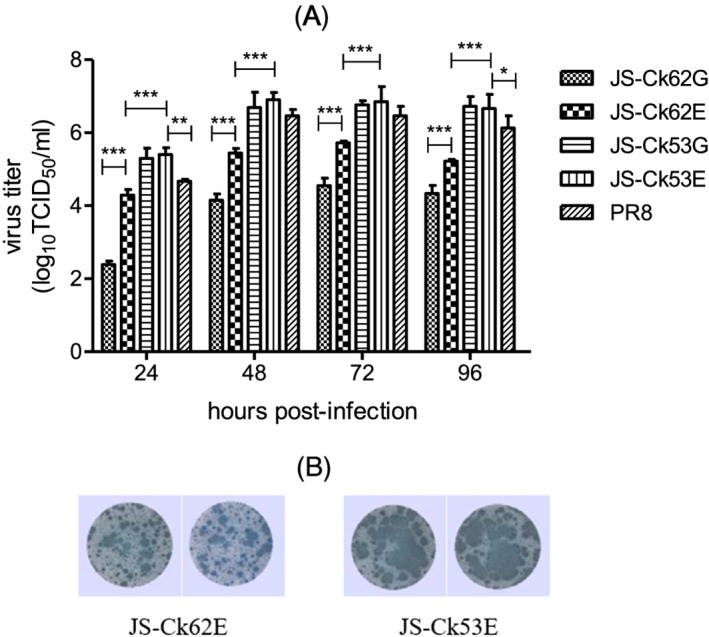
Growth characteristics of different viruses in MDCK cells. (A) Growth kinetics of different viruses. Cells were infected at 0.001 multiplicity of infection (MOI). Data are expressed as mean ± SD from three independent experiments and analyzed by a two‐way analysis of variance (ANOVA) using the GraphPad Prism 5 software (version 5.0. *, *P* < 0.05, **, *P* < 0.01, ***, *P* < 0.001). (B) Plaque formation ability of different viruses. Cells were infected at 0.01 MOI

We evaluated the virus replication ability in MDCK cells to compare the replication efficiency of viruses possessing different PB1 gene compositions. The results indicated that although JS‐Ck62E replicated significantly better than JS‐Ck62G (*P* < 0.001; Figure 1A), it grew significantly slower than JS‐Ck53G and JS‐Ck53E (P < 0.001; Figure 1A). No significant difference was observed between JS‐Ck53G and JS‐Ck53E. The growth capacity of JS‐Ck53G and JS‐Ck53E was comparable with the high‐yield virus PR8 at 48 and 72 h post‐infection and better than PR8 at 24 h (*P* < 0.01) and 96 h (*P* < 0.05) post‐infection (Figure 1A). JS‐Ck53E formed a larger plaque at the same MOI than the JS‐Ck62E virus meaning that JS‐Ck53E had a stronger growth phenotype than JS‐Ck62E (Figure 1B). The comparison results of replication ability based on hemagglutination assay between (6 + 2) and (5 + 3) of JS‐Hu were similar to that of JS‐Ck (Figure S1).

### 3.3 Effect of different PB1 on polymerase activity

**FIGURE 2:**
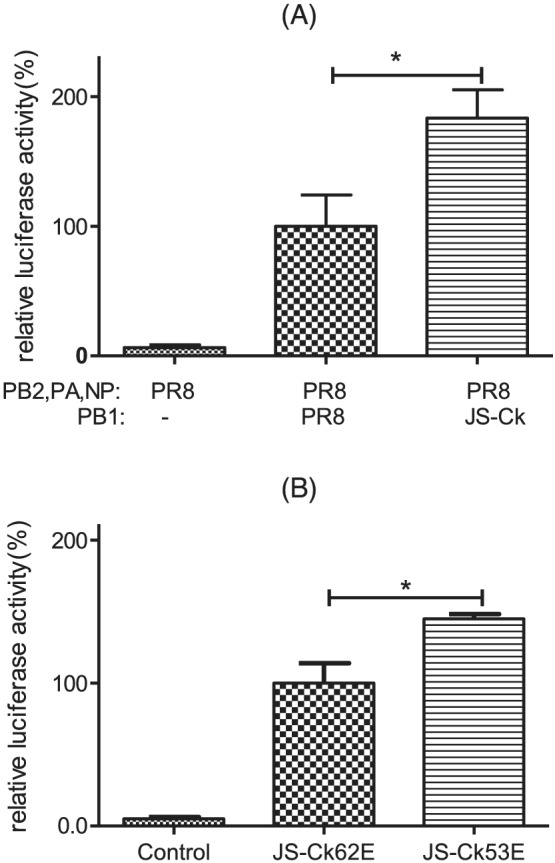
Comparison of the polymerase activity of different PB1 genes. (A) Polymerase activity measured by minigenome transfection; (B) polymerase activity measured by virus infection. Below the *X*‐axis are the components of the minigenome (A) and different viruses (B). Relative polymerase activities are shown as the ratio of firefly luciferase to Renilla luciferase activity. Renilla luciferase was the internal control for normalizing transfection efficiency. The background control was three plasmids transfected without the PB1 subunit in the minigenome transfection assay. Virus‐free culture media were the negative control in the virus infection assay. The relative luciferase activity associated with the minigenome of PR8 or JS‐Ck62E virus was set at 100%. Each experiment was repeated three times. Statistical significance was assessed using an unpaired two‐tailed *t* test with GraphPad Prism 5.0. (*, *P* < 0.05)

The combination minigenome containing the PB1 gene from JS‐Ck and PB2, PA, and NP from PR8 had higher polymerase activity than the combination with all four genes (PB2, PB1, PA, and NP) from PR8 (*P* < 0.05; Figure 2A). JS‐Ck62E and JS‐Ck53E viruses infected Vero cells after plasmid transfection. The results indicated that JS‐Ck53E had better polymerase activity than JS‐Ck62E (*P* < 0.05; Figure 2B). The above two results suggest that homologous PB1 substantially enhances polymerase activity.

### 3.4 Viral protein and HA protein yield

**FIGURE 3:**
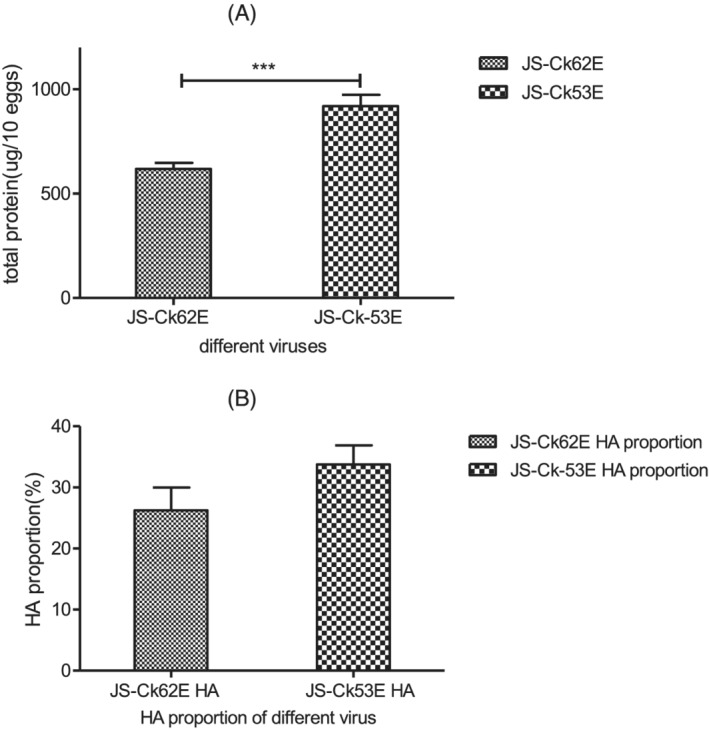
Protein yield of different viruses. (A) Viral total protein yield. (B) HA protein proportion. The HA proportion was determined using the following equation: HA1 + HA2/HA1 + HA2 + NP + M. Each experiment was repeated three times. Statistical significance was assessed using an unpaired two‐tailed t‐test with GraphPad Prism 5.0. (***, *P* < 0.001)

Egg‐propagated viruses of JS‐Ck62E and JS‐Ck53E were analyzed to examine the effect of PB1 on the yield of viral protein. The total protein content (per 10 eggs) of JS‐Ck53E was significantly higher than JS‐Ck62E (Figure 3A, P < 0.001). However, the proportion of HA protein in the total protein was not significantly different between JS‐Ck62E and JS‐Ck53E (Figures 3B and S2). The above two results manifested that the HA protein yield of the JS‐Ck53E virus is higher than JS‐Ck62E.

### 3.5 Identification of the antigenicity of different reassortant viruses

An HI assay was conducted using ferret antisera from JS‐Ck62G, JS‐Ck62E, and JS‐Ck53E to examine the influence of HA amino acid substitution on antigenicity. All viruses were immunogenic and induced HI antibody titers (320, 320, and 160, respectively) against homologous viruses. Each antiserum effectively inhibited all the tested RG H7N4 viruses. The difference in HI titer between the homologous and detected virus was below four‐folds. The substitution in HA (G to E) did not change the viral antigenicity, and all tested viruses exhibited similar antigenicity (Table 2).

**TABLE 2:** Antigenic analysis of candidate vaccine viruses by hemagglutination inhibition assay

Antigen ^a^	Ferret antisera		
	JS‐Ck62G	JS‐Ck62E	JS‐Ck53E
JS‐Ck62G	**320** ^b^	160	160
JS‐Ck62E	640	**320**	320
JS‐Ck53G	160	160	160
JS‐Ck53E	320	160	**160**
JS‐Hu62G	160	160	160
JS‐Hu62E	320	320	320

*Note*:

^a^ the source of antigen was presented in Table 1.

^b^ values in bold indicate homologous activity.

### 3.6 Pathogenicity of the JS‐Ck53E virus in cells and animals

JS‐Ck53E virus had various advantages, shown in the above results, and was selected for further pathogenicity tests in cells and animals. JS‐Ck53E plaques only occurred in the presence of trypsin treated with N‐tosyl‐L‐phenylalanine chloromethyl ketone (Figure 1B), indicating the low pathogenicity of the virus.^21^ Moreover, all ferrets survived during the two‐week observation period, with slight weight loss (<5%, data not shown). No other clinical symptoms (lethargy, respiratory signs and increased body temperature) were observed. In addition, viruses were only detectable in the respiratory system, including nasal wash fluids, nasal turbinates, and lungs but were undetected in other organs. The viral titer of nasal wash fluid, nasal turbinate, and lung was lower than the reference virus PR8, as previously reported for the same infection dose (Table 3).^19^

**TABLE 3:** Virus titer in different ferret organs

Virus	Animal no.	Nasal washes ^a^				Nasal turbinate ^b^	Lung ^b^	Other organs ^b^ ^,^ ^c^
		Day 1	Day 3	Day 5	Day7	Day 3	Day 3	Day 3
JS‐Ck53E	1	1.92	1.78	2.14	1.68			
	2	1.71	1.68	2.18	1.56			
	3					1.33	1.68	∕ ^d^
	4					1.67	∕	∕

*Note*:

^a^ log_10_TCID_50_/ml.

^b^ log_10_TCID_50_/g.

^c^ Other organs include the brain, spleen, intestine and olfactory bulb of the brain.

^d^ /, denotes no virus titer.

The virus (2048 hemagglutinin unit) was diluted in sterile PBS (1:10) and tested in the IVPI assay. All the chickens survived, and no clinical symptoms were observed during the 10‐day observation period. The IVPI of the virus was zero, thus, below the 1.2 mark.

Therefore, the reassortant JS‐Ck53E virus exhibited low pathogenicity and met the safety requirements as a CVV.

## 4 DISCUSSION

Vaccines are currently the most effective strategies for preventing and controlling influenza. Antigenic drift or shift influences the ability of viruses to escape pre‐existing immunity through mutations; thus, it is highly challenging to predict future epidemic influenza strains and develop a universal vaccine. Therefore, developing specific CVV with high replication ability and low pathogenicity remains crucial for vaccine production against the influenza virus, a potential cause of the next pandemic.

In this study, RG (5 + 3) and (6 + 2) viruses were constructed and assessed to investigate the suitable H7N4 CVV. Although (6 + 2) reassortant virus represents the most frequent gene composition in CVVs, mutations frequently occur in HA to achieve high yield characteristics.^19^, ^22^, ^23^, ^24^ Simultaneously, the occurrence of mutations carries the risk of inducing antigenic changes.^17^, ^25^ We hope introducing the homologous PB1 gene will improve the virus replication capacity and compensate for the mutation as previously reported.^7^, ^8^, ^10^ As anticipated, the results of replication ability indicated that (5 + 3) viruses were significantly better than (6 + 2) viruses. As a polymerase subunit, PB1 is critical for viral replication.^26^ The results of polymerase activity in both minigenome transfection and viral infection verified that homologous PB1 provided better polymerase activity than PR8 PB1. The better polymerase activity influenced high growth capacity of (5 + 3) virus, consistent with previous reports that avian PB1 improves polymerase activity better than human PB1.^27^, ^28^ In addition, some PB1 mutations are related to polymerase activity or replication ability.^12^, ^13^, ^29^, ^30^, ^31^, ^32^, ^33^ Most PB1 mutations were the same in PR8 and JS‐Ck, except for three motifs: 180G, 473 L, and 654N (in PR8 PB1) and 180E, 473V, and 654S (in JS‐Ck PB1) (Table S1). G180E and N654S improve growth ability in the three mutations, and V473L decreases polymerase activity and replication efficiency.^30^, ^33^ The results revealed that these motifs might contribute, in part, to the difference in growth ability or polymerase activity of viruses.

Furthermore, PB1 enhanced the growth ability of the (5 + 3) virus but had no visible correlation with G218E substitution in HA, which was inconsistent with a previous study.^11^ This substitution is prone to occur,^23^, ^34^ significantly improved the replication of the (6 + 2) virus but exhibited no significant effect on the (5 + 3) virus. Moreover, the (5 + 3) JS‐Hu virus showed increased replication than the corresponding (6 + 2) virus. Collectively, we speculate that the PB1 effect on growth capacity was superior to the HA substitution, but both factors lacked a superimposed enhancement effect. The mechanism needs further studies. An important feature of CVVs is high viral protein, especially HA protein yield. Directly PB1 increased the total protein yield and indirectly increased the content of HA protein. As previously reportsed,^23^, ^34^ the HA substitution at position 218 did not alter the antigenicity of these viruses, yetJS‐Hu had four amino acid differences in HA compared to JS‐Ck.^4^

Introducing avian PB1 in JS‐Ck53E sustained JS‐Ck53E low pathogenicity in chicken and ferrets. Position 66 of PB1‐F protein of JS‐Ck expressed N instead of S (Table S1), probably due to lack of mutation in N66S since S contributes to viral pathogenesis.^35^

The results of this study suggest that homologous PB1 is critical for enhancing the growth ability of the (5 + 3) virus without increasing virus pathogenicity. The JS‐Ck53E virus met the CVV requirements and is suitable for potential H7N4 influenza pandemic preparedness. However, the effect of homologous PB1 on the reassortant virus may depend on the strain, and different reassortant viruses need independent assessment.

## AUTHOR CONTRIBUTIONS

**Liqi Liu:** Data curation; formal analysis; methodology. **Zi Li:** Methodology; resources. **Jianfang Zhou:** Methodology; resources. **Jia Liu:** Data curation. **Xiyan Li:** Methodology. **Weijuan Huang:** Methodology. **Ning Xiao:** Methodology. **Dayan Wang:** Formal analysis; funding acquisition; project administration.

## CONFLICT OF INTEREST

The authors have no conflict of interest to declare.

## ETHICS STATEMENT

All animal experiments were performed following the guidelines for animal experiments described and approved by the Animal Care Welfare Committee of the National Institute for Viral Disease Control and Prevention, China CDC.

## Supporting information


**Table S1.** Compare key motifs between different PB1 gene
**Figure S1** Growth kinetics of different viruses measured by hemagglutination assay.
(A) RG JS‐Ck viruses; (B) RG JS‐Hu viruses. Data were expressed as mean ± SD from three independent experiments and analyzed by two‐way analysis of variance (ANOVA) using the GraphPad Prism 5 software (version 5.0. *, P < 0.05, **, P < 0.01, ***, P < 0.001).
**Figure S2 SDS‐PAGE profile of different viruses.** The same sample amount was used. The viral protein was deglycosylated using PNGase F at 37°C for 18 h, and then analyzed by SDS‐PAGE under reducing conditions. 62E:JS‐Ck62E, 53E:JS‐Ck53E, M: Marker (KD).

## Data Availability

Data are available on request from the corresponding author.
