# Supplementary material for: Homologous PB1 gene promotes the replication efficiency of avian influenza H7N4 candidate vaccine virus
Source: Influenza Other Respir Viruses. 2022 Jan 17;16(3):577–84. doi: 10.1111/irv.12954 (PMC8983892; doi:10.1111/irv.12954)

**Supporting information**

| **Table S1. Compare key motifs between different PB1 gene** | | | | | | |
| --- | --- | --- | --- | --- | --- | --- |
| Protein | Motif | PR8 | JS-Ck | Mutation | Phenotype | References |
| PB1 | 153 | N | N | D→N | Enhanced growth properties | 29 |
|  |  |  |  |  |  |  |
|  | 180 | **G** | **E** | G→E | Improved growth kinetics | 30 |
|  | 216 | S | S | S→G |  |  |
|  | 361 | S | S | S→R |  |  |
|  | 621 | Q | Q | Q→R |  |  |
|  | 654 | **N** | **S** | N→S |  |  |
|  |  |  |  |  |  |  |
|  | 296 | T | T | T→R | Enhanced polymerase activity and confer a virulent phenotype in mice | 31 |
|  |  |  |  |  |  |  |
|  | 447 | G | G | G→A | Enhanced polymerase activity | 32 |
|  | 621 | Q | Q | Q→R |  |  |
|  |  |  |  |  |  |  |
|  | 473 | **L** | **V** | V→L | Decreased polymerase activity and replication efficiency in mammalian cell | 33 |
|  |  |  |  |  |  |  |
|  | 577 | K | K | K→E | Increased Polymerase Activity and Pathogenicity in Mice | 12 |
|  | 622 | G | G | G→D | decreasing the polymerase activity and attenuating H5N1 virus virulence in mice | 13 |
|  |  |  |  |  |  |  |
| PB1-F2 | 66 | N | N | N→S | Enhanced virulence in mice | 35 |
| Note: Different amino acids were in bold. | | | | | | |

**Legends to Figure S**

**Fig.S1** **Growth kinetics of different viruses measured by hemagglutination assay.**

(A) RG JS-Ck viruses; (B) RG JS-Hu viruses. Data were expressed as mean ± SD from three independent experiments and analyzed by two-way analysis of variance (ANOVA) using the GraphPad Prism 5 software (version 5.0. *, P<0.05, **, P<0.01, ***, P<0.001).

**Fig.S2 SDS-PAGE profile of different viruses.** The same sample amount was used. The viral protein was deglycosylated using PNGase F at 37°C for 18 h, and then analyzed by SDS-PAGE under reducing conditions. 62E:JS-Ck62E, 53E:JS-Ck53E, M: Marker (KD).

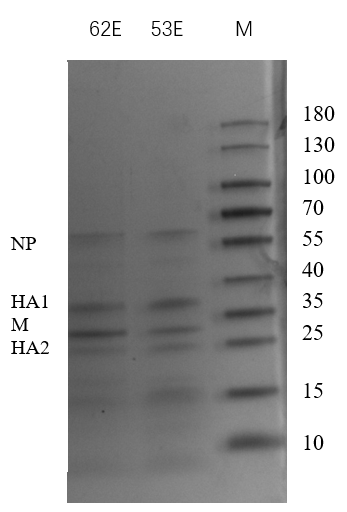

Supplement: Supplementary file 1 — Table S1. Compare key motifs between different PB1 gene Figure S1 Growth kinetics of different viruses measured by hemagglutination assay. (A) RG JS‐Ck viruses; (B) RG JS‐Hu viruses. Data were expressed as mean ± SD from three independent experiments and analyzed by two‐way analysis of variance (ANOVA) using the GraphPad Prism 5 software (version 5.0. *, P < 0.05, **, P < 0.01, ***, P < 0.001). Figure S2 SDS‐PAGE profile of different viruses. The same sample amount was used. The viral protein was deglycosylated using PNGase F at 37°C for 18 h, and then analyzed by SDS‐PAGE under reducing conditions. 62E:JS‐Ck62E, 53E:JS‐Ck53E, M: Marker (KD). [file IRV-16-577-s001.docx]
